# Porous Materials Confining Single Atoms for Catalysis

**DOI:** 10.3389/fchem.2021.717201

**Published:** 2021-07-21

**Authors:** Tao Zhu, Yiwei Han, Shuai Liu, Bo Yuan, Yatao Liu, Hongli Ma

**Affiliations:** ^1^ Institute of Atmospheric Environmental Management and Pollution Control, China University of Mining & Technology (Beijing), Beijing, China; ^2^ State Key Laboratory of Organic Geochemistry, Guangzhou Institute of Geochemistry, Chinese Academy of Sciences, Guangzhou, China

**Keywords:** porous materials, single-atom catalysts, support, confining, zeolite

## Abstract

In recent years, single-atom catalysts (SACs) have received extensive attention due to their unique structure and excellent performance. Currently, a variety of porous materials are used as confined single-atom catalysts, such as zeolites, metal-organic frameworks (MOFs), or carbon nitride (CN). The support plays a key role in determining the coordination structure of the catalytic metal center and its catalytic performance. For example, the strong interaction between the metal and the carrier induces the charge transfer between the metal and the carrier, and ultimately affects the catalytic behavior of the single-atom catalyst. Porous materials have unique chemical and physical properties including high specific surface area, adjustable acidity and shape selectivity (such as zeolites), and are rational support materials for confined single atoms, which arouse research interest in this field. This review surveys the latest research progress of confined single-atom catalysts for porous materials, which mainly include zeolites, CN and MOFs. The preparation methods, characterizations, application fields, and the interaction between metal atoms and porous support materials of porous material confined single-atom catalysts are discussed. And we prospect for the application prospects and challenges of porous material confined single-atom catalysts.

## Introduction

The particle size of single-atom catalysts (SACs) has reached the minimum limit and has an atomic utilization rate close to 100%. In recent years, they have been widely researched and applied in the fields of electrocatalysis, photocatalysis and energy conversion (Ding et al., 2019; Samantaray et al., 2020). Single-atom catalysts exhibit excellent activity, selectivity and stability in some reactions, and their unique properties are closely related to the metal-support interaction (Wang et al., 2017).

The support plays a key role in the catalytic performance of single-atom catalysts, such as the strong interaction between metal and support (Kunwar et al., 2019; Qiao et al., 2020), the induction of charge transfer between metal and support, the inhibition of the electronic structure of support metal, and the influence of the adsorption energy of reaction intermediates, which ultimately improve the performance of the catalyst (Flytzani-Stephanopoulos and Gates, 2012; Yan et al., 2019). The support affects the uniformity of atom dispersion (Park et al., 2019), the trapping and stabilization of single metal atoms by support defects, and the influence of the coordination environment of the support on the catalytic activity and selectivity (Tang et al., 2020). Different types of support supported single-atom catalysts exhibit different catalytic performance. The interaction between metal atoms and the support controls the catalytic performance of SACs (Lou and Liu, 2017; Ishida et al., 2020). Common types of supports include metal oxides [Al_2_O_3_ nanorods (Cui et al., 2017), FeO_x_ (Qiao et al., 2011) and CO_3_O_4_ (Lou et al., 2020), etc.], metal organic frameworks (MOF) (Otake et al., 2018; Zhang et al., 2019a), Ti_3_C_2_T_x_MXene nanosheets (Bao et al., 2021), carbon materials [graphene (RGO) (Chen et al., 2019), carbon nanotubes (CNT) (Cheng et al., 2019) and carbon nitride (CN) (Chen et al., 2018) etc.] and zeolites (Fang et al., 2020; Zhang et al., 2020a) and so on.

Single-atom catalysts confined in porous materials have unique characteristics. First, porous materials usually have a huge specific area, which can provide more attachment sites for metal atoms (Yang et al., 2019). Secondly, the pore structure of porous materials can prevent the aggregation of metal atoms (Qin et al., 2018) and increase the loading of metal atoms. Thirdly, the porous material has high thermal stability and adjustable acidity, which is convenient for the microenvironment control of the metal atom and the support (Zhang et al., 2020b).

Here, we reviewed recent studies on single-atom catalysts confined in porous materials, mainly zeolites, carbon nitride (CN), and MOFs. We pay attention to the preparation methods, characterizations, catalytic mechanism and practical application of single atom catalysts confined within porous materials.

## Single Atoms Confined in Zeolites

**FIGURE 1:**
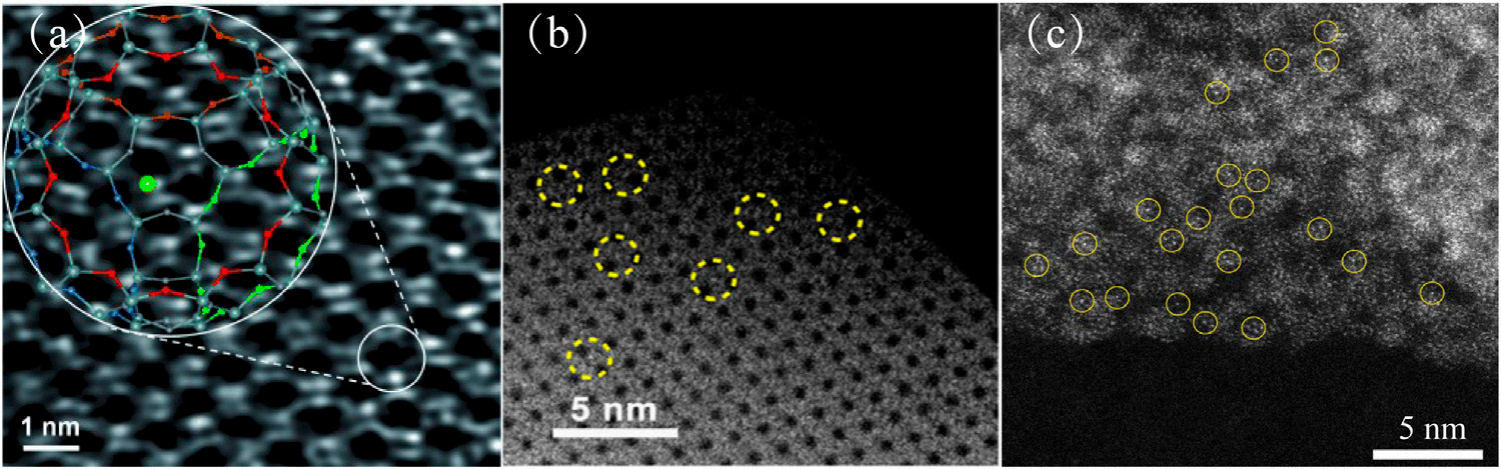
**(A)** Aberration-corrected HAADF-STEM images of the sample prepared by adsorption of Au(CH_3_)_2_ (acac) in zeolite NaY (Lu et al., 2012). Copyright 2012, Wiley. **(B)** AC-HAADF-STEM images of Ru SAs/S-1 (Qiu et al., 2019). Copyright 2019, American Chemical Society. **(C)** HAADF-STEM image of Pt-SA-Ce-MOF shows that Pt atoms (in yellow circle) were uniformly dispersed in the Ce-MOF support (Guo et al., 2020). Copyright 2020, American Chemical Society.

Zeolites are an important porous material with a wide range of applications, usually composed of alumina and silica tetrahedrons. They have a regular network structure. Metal-zeolite composites are widely used in thermocatalysis (Zhang et al., 2020a) and dehydrogenation/hydrogenation (Liu et al., 2019a). Metal-zeolite composites have been extensively researched and successfully prepared by a variety of methods, such as postencapsulation methods, *in situ* encapsulation methods or zeolite-shell-encaged methods (Kosinov et al., 2018; Wang et al., 2019a). Zeolites have a uniform pore structure and cage structure. Metals can be uniformly dispersed in the pores and cage structures of the zeolites, which can confine the metal atoms firmly (Shamzhy et al., 2019). These metal atoms can be directly observed using aberration-corrected high-angle annular dark-field scanning transmission electron microscopy (AC-HAADF-STEM) (Ortalan et al., 2010; Lu et al., 2012; Qiu et al., 2019). Lu et al. reported that the AC-HAADF-STEM images of metal atoms show bright dots that are distinct from the zeolite (Figure 1A) (Lu et al., 2012); this has also been observed in other studies (Figures 1B,C). At the same time, transmission electron microscopy (TEM) and scanning transmission electron microscopy (STEM) showed that subnanometer clusters and nanoparticles were not observed on the zeolites (Qiu et al., 2019).

**FIGURE 2:**
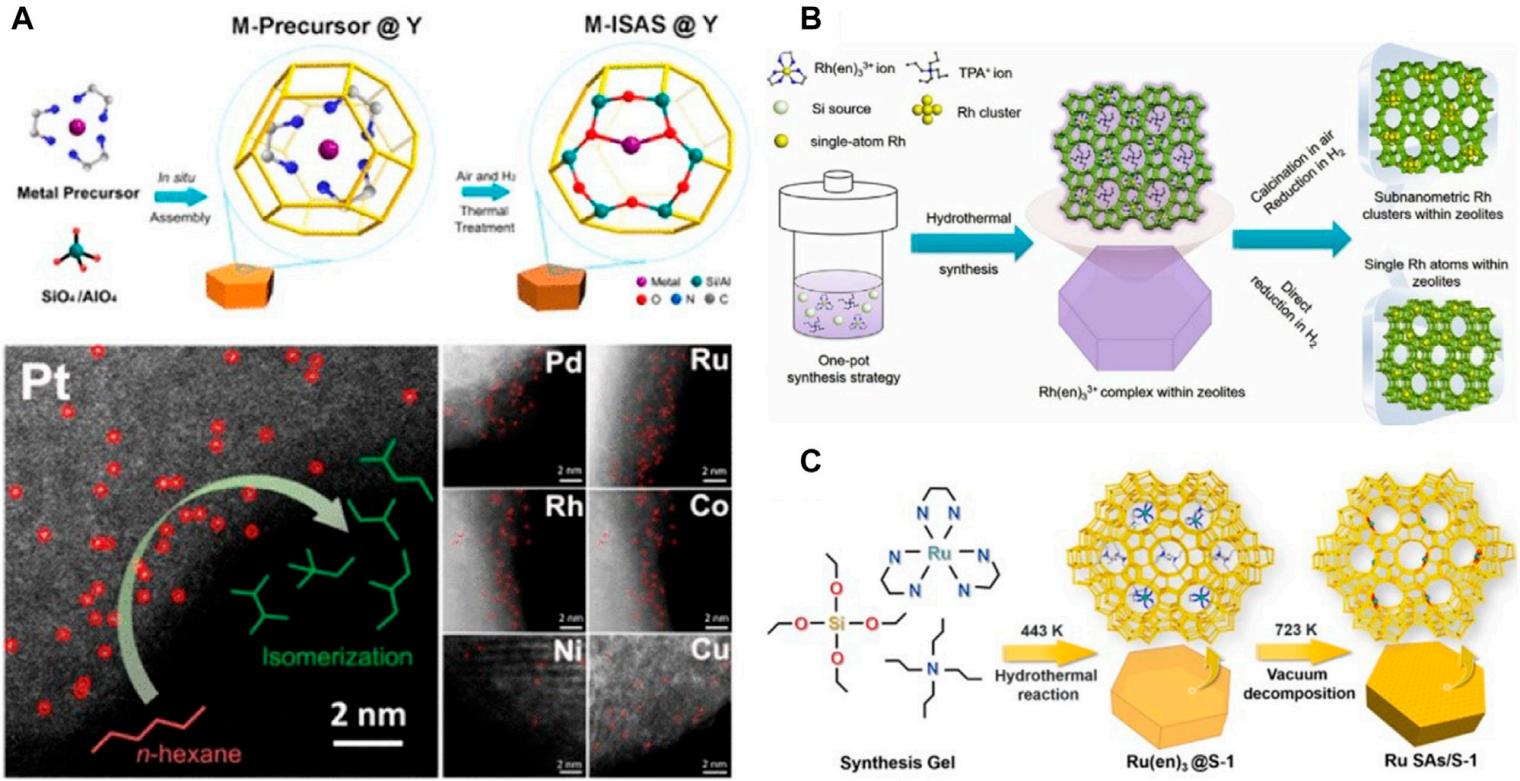
Schematic illustration synthesis and characterization of SACs. **(A)** *In situ* separation and confinement of a metal precursor in a *β*-Cage and characterization of M-ISAS@Y (Liu Y. et al., 2019). Copyright 2019, American Chemical Society. **(B)** Schematic illustration of the fabrication of the Rh@S-1 catalysts (Sun et al., 2019). Copyright 2019, Wiley **(C)** Synthesis Procedure of Ru SAs/S-1 (Qiu et al., 2019). Copyright 2019, American Chemical Society.

Although significant progress has been made in SACs within zeolites, their controllable synthesis and characterization are still a challenge (Wang et al., 2018). A method of *in-situ* synthesis in the zeolite crystallization process was used to prepare single-atom catalysts confined in the zeolites, and the metal and ethylenediamine were complexed to form a precursor to prevent metal precipitation and aggregation. This method is universal and has been successfully applied to Co, Rh, Pd, Ni, Pt, Cu SACs (Figure 2A) (Liu Y. et al., 2019). Aberration-corrected high-angle annular dark-field scanning transmission electron microscopy (AC-HAADF-STEM) images (Figure 2A) show metal single atoms as bright dots that are distinct from the zeolite. Compared with the impregnation method, due to the limitation of micropore diffusion, the precursors are usually gathered in the shallow layer of the zeolite crystals. The *in-situ* synthesis strategy ensures the uniform dispersion of the precursors in the entire block. Density functional theory (DFT) calculations and extended X-ray absorption fine structure (EXAFS) have confirmed that metal atoms were coordinated with two oxygen atoms in Al-O-Si bridges and the six rings of the zeolite Y framework for Pt atoms was the most stable configuration (Figure 2A). The Pt sites from both *β*-cages and supercages of Y zeolite were beneficial for the catalytic interaction.

Similarly, Sun et al. encapsulated rhodium atoms within MFI silicalite-1 (S-1) and ZSM-5 zeolites by using [Rh(NH_2_CH_2_CH_2_NH_2_)_3_]Cl_3_ complex as a precursor under one-pot hydrothermal synthesis conditions followed by H_2_ reduction (Figure 2B) (Sun et al., 2019). The Ru SAC (Ru SAs/S-1) confined in S-1 load was successfully prepared by the strict control of the detemplate and the demamination atmosphere for the first time (Figure 2C) (Qiu et al., 2019). Extended X-ray absorption fine structure (EXAFS) analyses show that all zeolite-encaged Rh species possess a higher oxidation state than the Rh foil because of the formation of superfine Rh species. Most significantly, the zeolite-encaged SACs exhibited superior efficiency in tandem hydrogenation of nitroarenes by coupling with AB hydrolysis. Unlike the *in-situ* synthesis, Shan et al. anchored the Rh atoms in ZSM-5 supports using a heat treatment protocol (Shan et al., 2017). Under mild conditions, Rh SACs was used to catalyze the direct conversion of methane to methanol and acetic acid in the presence of oxygen and carbon monoxide. The yield of acetic acid was about 22,000 μmol/g catalyst, and the selectivity was 60–100%.

**FIGURE 3:**
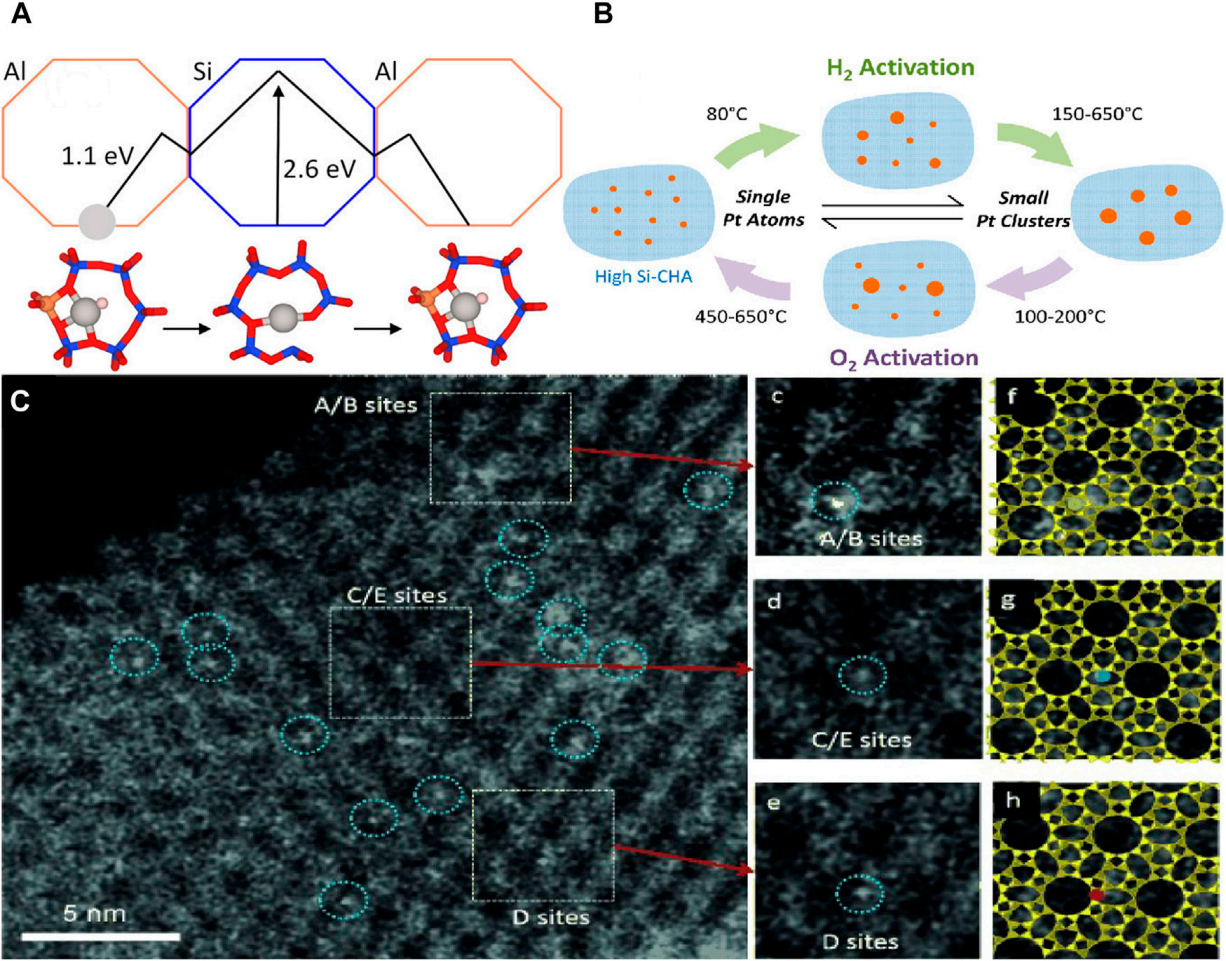
**(A)** Diagram of positional migration and effective energy of metal atoms in zeolite (Hou et al., 2020). Copyright 2020, American Chemical Society. **(B)** Conversion of catalysts in different atmospheres (Moliner et al., 2016). Copyright 2016, American Chemical Society. **(C)** STEM images showing site-isolated Pt atoms in KLTL zeolite in the as-prepared samples (Kistler et al., 2014). Copyright 2014, Wiley.

The investigation of the stability mechanism of metal atoms in zeolites is still a challenge. Hou et al. identified the locations and energetic barriers of ultrasmall Pt metal particles within the LTA zeolites by an unbiased density functional global optimization strategy (Hou et al., 2020). The authors suggested that the six-membered ring in zeolite is the optimal structure for stabilizing Pt atoms, and the formed O-Pt-O structure maximizes the bonding of Pt and the transfer of charges, so that the escape of Pt requires a high potential barrier, which may be the reason why the catalyst resists sintering (Figure 3A). Other work revealed for the first time the location of the Rh atoms within 5-membered rings (MRs) of MFI which are stabilized by zeolite framework oxygens (Qiu et al., 2019). This result is consistent with Pt atoms located firmly in zeolite Y (Liu Y. et al., 2019). Density functional theory (DFT) calculations and extended X-ray absorption fine structure (EXAFS) fitting showed that Pt was coordinated with two oxygen atoms on Al-O-Si in zeolites to form Pt-O bonding. Such strong bonding to six-ring sites endows Pt SACs with excellent durability against metal sintering in zeolites. The authors also found that Al atoms were important to the stability of SACs because of strong interaction between Pt and O in Al-O-Si structure. The Pt sites within supercages and *β* cages were beneficial for contact between reactant and Pt sites. At the same time, Moliner et al. also reported that Pt atoms within high-silica CHA zeolites have stability toward metal sintering (Moliner et al., 2016). Moreover, there is a very interesting founding that the particle size of Pt nanoparticles can be reversibly transformed under hydrogen and oxygen and different temperature conditions, ranging from single metal atoms to ∼1 nm (Figure 3B). Similarly, Pt atoms in zeolite LLTL were observed by XAS and AC HAADF-STEM, which showed the location of site-isolated Pt atoms within zeolite (Figure 3C). But not all Pt SACs supported in zeolites have high activity. For example, studies have shown that sub-nano Pt clusters are more active than single Pt atoms in the low-temperature CO + NO reaction (Fernández et al., 2019).

Mo SACs confined in zeolites for CH_4_ conversion have also been reported (Konnov et al., 2020). A single-site Mo-containing nanosized ZSM-5 zeolites was prepared by a novel “top-down” synthesis approach with sodium molybdate self-pressurized calcination of nano-zeolite. This Mo SAC exhibited thermal stability up to 1,000°C and excellent performance in the conversion of CH_4_ to H_2_ or higher hydrocarbons. In addition, the dispersion of metal atoms was maintained even in steam and high temperature environment. It is proposed that the distinctive performance of Mo SAC is the result of the introduction of Mo atoms in the zeolite framework, which can heal silanol defects to severe structural degradation. Similarly, Dubray et al. also found that the substituting molybdenum for skeleton silicon can significantly reduce the defect content of silanol and make the zeolites with high hydrophobicity (Dubray et al., 2019).

Because of their wide application, zeolite-confined SACs has aroused great interest among researchers. Liu et al., 2020a used one-pot synthesis approach to anchor Ir single atoms in pure-silica MWW zeolite (Liu et al., 2020a), BEA zeolite (Jaegers et al., 2019)or in zeolite Y (Bayram et al., 2015) and these Ir SACs were used in the alkane hydrogenolysis reaction (Liu et al., 2020a), ethylene polymerization reaction (Jaegers et al., 2019), and cyclohexene hydrogenation reaction (Bayram et al., 2015). HAADF-STEM imaging and extended X-ray absorption fine structure (EXAFS) analysis, the authors proposed that reduction temperature and average particle sizes have a great influence on the stability and activity of metal atoms (Ortalan et al., 2010; Bayram et al., 2015; Liu et al., 2020a). Ir clusters below 0.7 nm are the best size for propane hydrogenolysis (Liu et al., 2020a). Ir SACs also showed excellent performances during the butadiene upon reaction with ethylene under mild conditions (80–220°C, 1°bar)(Jaegers et al., 2019). Furthermore, subnanometric metal species (single atoms and clusters) Pt or Pt-Sn species confined in zeolites (L. Liu et al., 2020c; Liu et al., 2020a) or site-isolated BO_3_ units confined in MCM-22 zeolites (Altvater et al., 2020) were also reported to be the catalytically active sites in the dehydrogenation of propane to form propylene (Liu L. et al., 2019; Liu et al., 2020a) or oxidative dehydrogenation of propane to propene (Altvater et al., 2020), respectively.

The results described above show that the regular pore structure in zeolites is conducive to confine metal single atoms to prevent their aggregation. Zeolites-confined SACs exhibit high activity and stabilities. The *in-situ* synthesis method simplifies the synthesis steps and provides a method for large-scale preparation (Wang et al., 2016), but the metal loading is low, and the thermal stability needs to be further studied. In any case, the above research provides a successful case for other types of zeolites confined single-atom catalysts. Although the preparation of high-temperature-resistant zeolites confined single-atom catalysts is still a challenge, zeolites have high thermal stability and great potential in thermal catalysis.

## Single Atoms Confined in Carbon Nitride (CN)

Doping of N into the carbon network, which changes the electronic properties of carbon, and make carbon nitride have visible light response property, excellent stability and biocompatibility (Li et al., 2020). CN materials are widely used in electrocatalysis (Gao et al., 2020; Zhou et al., 2020) and photocatalysis (Li et al., 2020). At present, carbon nitride (CN) is one of the most studied catalysts with various forms including carbon nanotubes (CNTs) (Feng et al., 2020), porous nitrogen-doped carbon nanowires (NCNWs) (Li et al., 2019b). Carbon nitride is considered to be an ideal support for SACs due to its tri-s-triazine structure, in which the N/C coordination network can be used to anchor metal atoms.

**FIGURE 4:**
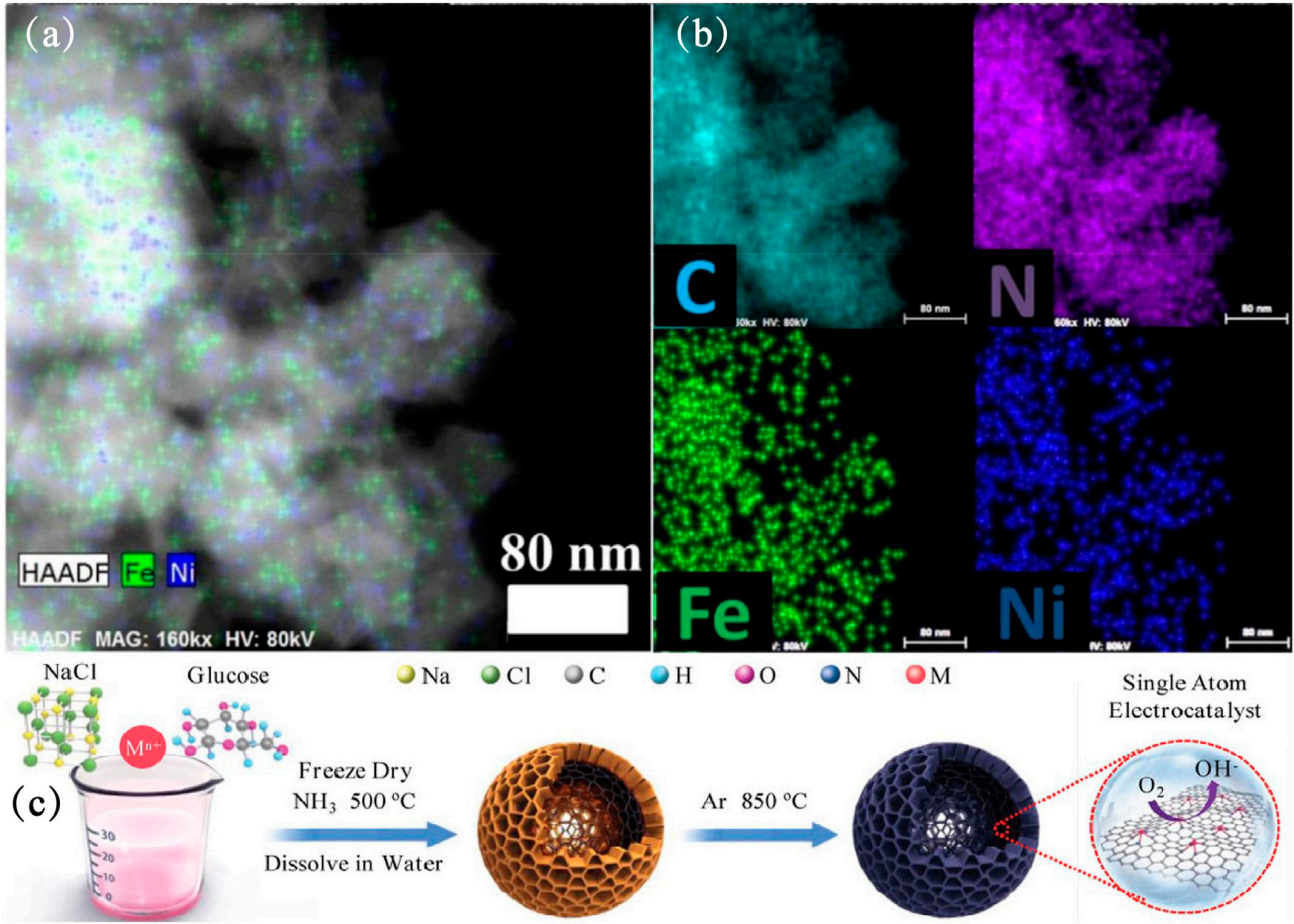
**(A,B)** HAADF-STEM of FeNi-N_6_ and corresponding element mapping patterns of FeNi-N_6_ (Zhou et al., 2020). Copyright 2020, American Chemical Society. **(C)** Schematic illustration for preparing M1-HNC-500-850 (Zhang et al., 2020c). Copyright 2020, Wiley.

The work of CN in the preparation of single-atom catalysts by confining Fe atoms has recently been reported. Li et al., 2019a used a facile secondary atom assisted approach to anchor Fe atoms in the porous nitrogen-doped carbon nanowires (NCNWs) (Li et al., 2019b), honeycomb-like nitrogen-doped carbon (Zhang et al., 2020c) or N-decorated mesoporous carbonaceous spheres (Chen et al., 2020a), and these Fe SACs present excellent catalytic performances (Chen et al., 2020b; Li et al., 2019b), superior stability (Wei et al., 2020). The Fe and Ni atoms was observed as a dual dot form, which is uniformly dispersed in the carbon skeleton by High-angle annular dark-field spherical aberration correction scanning electron microscope (HAADF-STEM) (Figures 4A,B). DFT calculations demonstrated that the high ORR activity was attributed to highly efficient single-atom Fe sites decreasing the energy barriers in multi-step electron transfer process (Li et al., 2019b).

Zhang et al. used NaCl as a template and utilized the chelation effect of glucose to adsorb transition metal ions (Fe/Co/Ni) onto a thin layer wrapped on the surface of NaCl. Finally, after two steps of calcination, honeycomb-like transition metal electrocatalysts were obtained (Figure 4C) (Zhang et al., 2020c). The as-made electrocatalyst with Metal-N_4_ active sites exhibits outstanding electrochemical performance for ORR, com-parable to that of commercial Pt/C. Among the transition metal (Fe/Co/Ni), Fe-N_4_ active sites has the best performance. Density functional theory (DFT) calculations showed that the strong p-d coupling effect induced by Fe-N_4_ combines active electrons in the form of van-Hove singularities, which promotes the electrical activity of the d band. This is beneficial to the electron transfer related to the Fe-3d band of ORR. Similar metal and N-coordination structures (Ag-N_4_) show remarkable NH_3_ yields in electrochemical ammonia synthesis applications (Chen et al., 2020b).

COF-absorption-pyrolysis strategy was reported to anchor metal atoms on COF-derived nitrogen-doped carbon nanospheres for ORR reactions in alkaline media (Wei et al., 2020). The experimental results showed that the activity of the Fe SACs prepared by this method were better than that of commercial 20 wt% Pt/C, and exhibited excellent stability and methanol resistance. Meanwhile, the authors prepared the Co and Ni SACs with the same method, demonstrating the universality of the proposed method.

**FIGURE 5:**
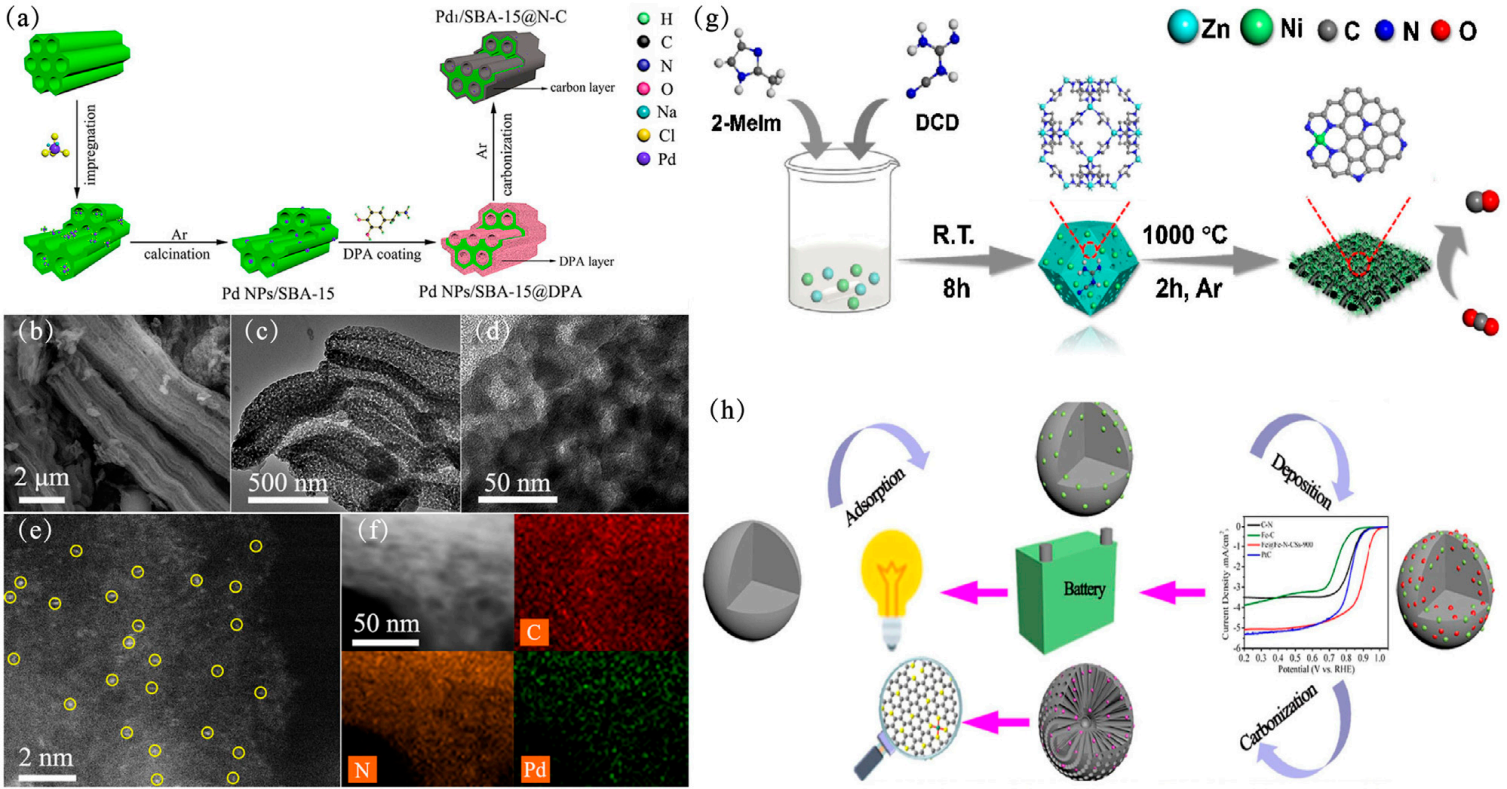
The construction and characterization of Pd_1_/SBA-15@N-C (a-f) (Li et al., 2021). Copyright 2021, American Chemical Society. **(A)** Synthetic procedure of Pd_1_/SBA-15@N-C. **(B)** SEM, **(C)** TEM, **(D)** HR-TEM, and **(E)** AC HAADF-STEM images of Pd_1_/SBA-15@N-C. **(F)** Elemental mapping of Pd_1_/SBA-15@N-C. **(G)** synthesis procedure of Fe at Fe-N-CSs electrocatalyst (Lu et al., 2019). Copyright 2019, Elsevier. **(H)** Scheme for the synthesis of Ni SAs/NCNTs (Chen et al., 2020b). Copyright 2020, Elsevier.

Although many high-performance single-atom catalysts have been synthesized, large-scale synthesis of SACs within CN is still a challenge. Furthermore, to meet industrial needs, Zhao et al. developed a cascade anchoring strategy to prepare of a series of metal-NC SACs on a large scale. The metal loading of metal-NC SACs for CO_2_ reduction are up to 12.1 wt% (Zhao et al., 2019a). Recently, Li et al., 2019b synthesized a Pd_1_/SBA-15@N-C SAC using thermal carbon atomization strategy (Li et al., 2021). A layer of dopamine is coated on the SBA-15 zeolite, a core-shell structure is finally formed after carbonization, and the Pd atoms are anchored on the nitrogen-doped carbon shell on the SBA-15 support (Figure 5A). As shown in Figures 5B,C, dopamine hydrochloride (DPA) is transformed into a porous N-doped core-shell during the carbonization process, and SBA-15 maintains its original shape. High-resolution transmission electron microscopy (HR-TEM) (Figure 5D) showed that no nanoparticles or clusters were formed on SBA-15@N-C, which proved that metal Pd existed in the form of single atoms on the support to some extent (Zhang et al., 2019b). Through aberration-corrected high-angle annular dark-field scanning transmission electron microscope (AC HAADF-STEM) (Figure 5E), it can be clearly seen that Pd atoms are uniformly distributed on the carrier instead of nanoparticles or nanoclusters, which is consistent with HR-TEM. Elemental mapping of C, N, Pd also proved that Pd atoms are evenly dispersed on the support (Figure 5F). In the selective hydrogenation of phenylacetylene, the TOF value of N-C is as high as 12,060°h^−1^, and the conversion and selectivity of styrene are 96 and 93%, respectively. In contrast, Pd NPs/SBA-15 had only 2,113°h^−1^ TOFs in this reaction. The authors suggested that a N-doped carbon layer on the SBA-15, dispersed Pd atoms, unique coordination environment and special catalytic mechanism are possible reasons for these results. Furthermore, XAFS analysis indicated that each palladium atom was coordinated with four nitrogen atoms on the N-doped carbon layer. Chen et al. developed a rational designed carbonaceous spheres templated strategy to prepare highly efficient ORR electrocatalysts (Figure 5G) (Chen et al., 2020b). The obtained catalyst had high stability and resistance to methanol due to desired specific surface area and meso/macroporous peculiarity, which is beneficial to mass transfer during the ORR process.

**FIGURE 6:**
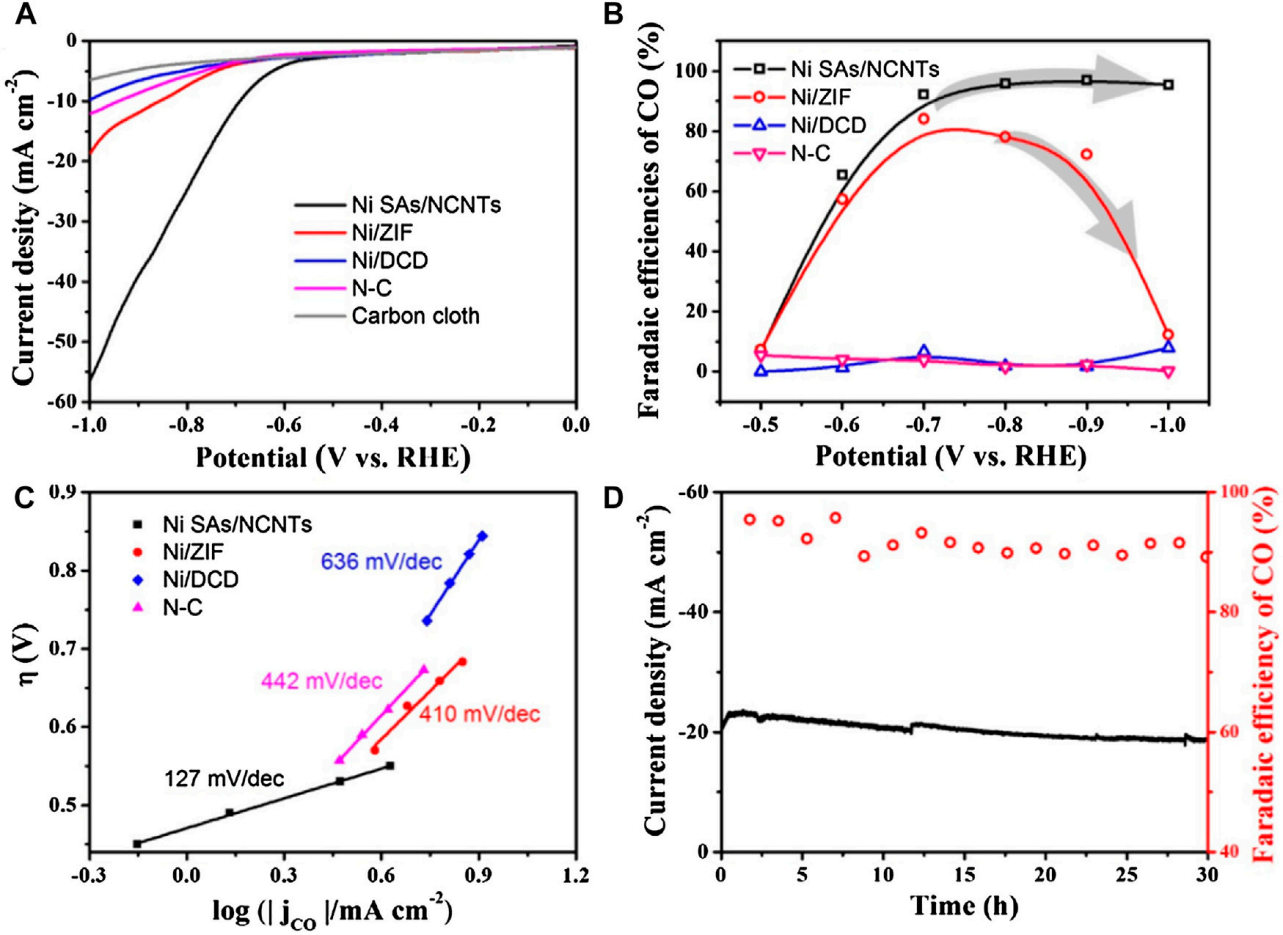
**(A)** Linear sweep voltammetric curves, and pure carbon cloth servers as background. **(B)** Faradaic efficiencies of CO at different applied potentials. **(C)** Tafel plots for producing CO. **(D)** Catalytic stability test of Ni SAs/NCNTs at −0.75 V for 30 h (Lu et al., 2019). Copyright 2019, Elsevier.

Ni SACs confined in carbon nanotubes for efficient electrochemical CO_2_ reduction have also been reported (Lu et al., 2019). This catalyst with a Ni loading as high as 6.63 wt% was prepared by pyrolysis of the mixture of small organic molecules with Zn/Ni salts (Figure 5H). As shown in Figure 6A, compared to Ni/ZIF and Ni/DCD, Ni SACs exhibited excellent activity. When carbon dioxide was electrically reduced to carbon monoxide (CO) in a wide potential range of −0.7 to −1.0°V, the high Faraday efficiency was about 95%, and the current density was 57.1 mAcm^−2^ compared with the reversible hydrogen electrode (RHE) (Figure 6B) because of its higher surface area. While for the other catalysts, they shared the most sluggish activity at high potential range from −0.8 to −1.0 V (Figure 6B). The Ni SAs/NCNTs exhibited the lowest Tafel slope (127 mV/dec), which is smaller than that of other catalysts (Figure 6C), indicating favorable kinetics for the formation of CO. The catalysts had high stability, and the faradaic efficiency (FE) for CO and the current density keep them stable during 30 h operation (Figure 6D).

Single-atom catalysts confined in carbon nitride (CN) have been extensively studied. Among them, the doped N element is of great significance for anchoring metal atoms. In CN-confined SACs, there are usually 3–4 N atoms coordinated with metal atoms and act as catalytic active centers, showing excellent performance. A more efficient and simple preparation method to prepare CN-confined single-atom catalysts on a large scale is of great significance for industrial applications. Micro environment regulation for confined in NC monatomic catalyst performance plays an important role (Han et al., 2021). Furthermore, graphitic carbon nitride (g-C_3_N_4_) and graphene porous materials exhibit unique properties as single-atom catalyst carriers (Han et al., 2019; Lv et al., 2020; Zhao et al., 2020). Adding nitrogen and other elements (such as B) to CN at the same time helps to improve the catalytic activity (Peng et al., 2020). The diatomic sites of the confinement on CN play a catalytic and synergistic role, which has gradually attracted the attention of researchers (Han et al., 2020). The single-atom catalysts confined in these materials have gradually attracted the attention of researchers.

### Single Atoms Confined in Metal Organic Frameworks (MOFs)

Metal organic frameworks (MOFs) are a class of porous crystal solid materials, which are promising highly dispersed metal catalyst support materials. This is largely due to their ability to introduce metal species into tunable inorganic metal nodes and organic ligands, thus providing coordination sites for metal atoms (Mendonca and Snurr, 2019; Cui et al., 2020). For example, Fe SACs confined in MOF were applied to cancer phototherapies (L. Wang et al., 2019b), nanozymes (Niu et al., 2019), and electrocatalysts for proton-exchange membrane fuel cells (PEMFCs) (Liu et al., 2018).

**FIGURE 7:**
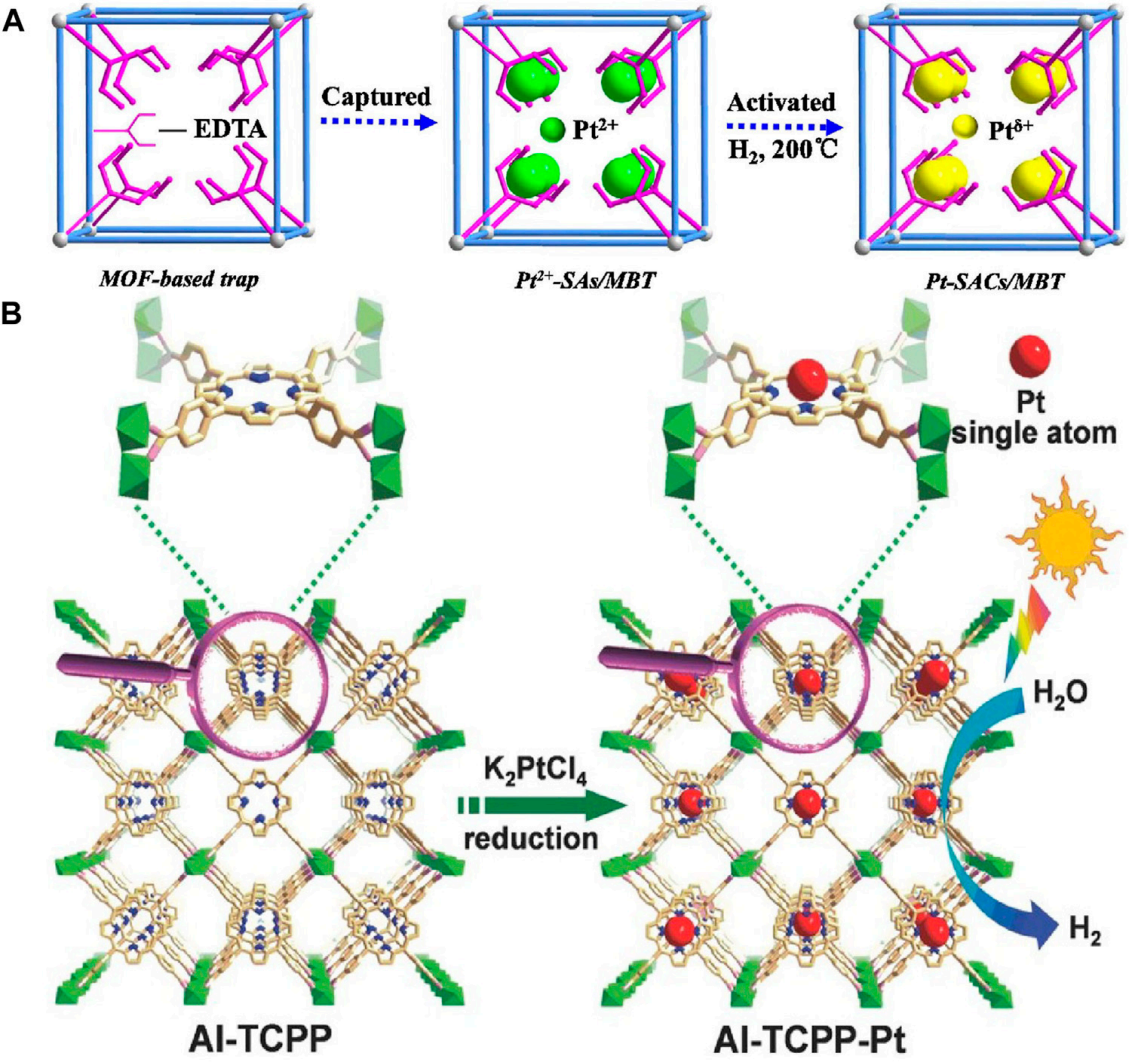
**(A)** Schematic illustration of single-atom Pt catalysts encapsulated in the MOF-808-EDTA *via* single metal ion trap method (Li et al., 2019a). Copyright 2019, Elsevier. **(B)** Schematic illustration showing the synthesis of Al-TCPP-Pt for photocatalytic hydrogen production (Fang et al., 2018). Copyright 2018, Wiley.

A general synthesis platform has been developed for a stable single-atom catalyst in a metal-organic framework (MOF) structure (Li et al., 2019a). Using the MOF-808-based single ion capture method, ethylenediaminetetraacetic acid (EDTA) ligand exchanged with the original formate ligand anchored on the metal node of the Zr_6_ cluster, effectively capturing a single metal ion (Figure 7A). The synthesized MOF-808-EDTA coated monoatomic platinum catalyst has excellent photocatalytic hydrogen evolution activity (68.33 mmol g^−1^H^−1^) and high stability. At 420 nm, the apparent quantum efficiency reaches 67.6%. DFT calculations showed that the excellent performance was attributed to the lower hydrogen binding free energy, which Pt atoms effectively made under photocatalytic conditions.

Different from the method of Li’s research (Li et al., 2019a), Fang et al. used Al-TCPP to reach single Pt atoms in 3D microporous framework of MOF based on the strong interaction between pyrrolic N atoms and Pt atoms (Figure 7B) (Fang et al., 2018). The catalyst exhibited significant hydrogen evolution activity in the photocatalytic water splitting reaction for hydrogen production, and its conversion frequency was 35 h^−1^, which was about 30 times that of nanoparticles. DFT calculations showed that the incorporation of single platinum atoms into MOF improved the binding energy of MOF, which greatly improved the photocatalytic activity for hydrogen production.

In order to clarify the coordination principle of the active center composed of a metal atom and several coordination anions, Liu et al., 2020b employed density function theory (DFT) calculations revealed that the oxygen reduction reaction (ORR) activity of SACs depended on the match between the OH* adsorption on metal atoms and the electronegativity of the coordination anion, and the stronger the OH* adsorption, the higher the electronegativity required for the coordination anion (Liu et al., 2020c).

Defects in MOF are beneficial to anchor metal atoms, Guo and coworkers reported that Pt atoms were anchored by defects in Ce-MOF (Guo et al., 2020). The strong coupling between isolated Pt atoms and ceria in MOF provided abundant active sites for CO conversion. In other work, Cu atoms were anchored by the defects of zirconia clusters in the metal organic framework UiO-66 to prepare a single-atom catalyst (Abdel-Mageed et al., 2019), which has high stability and activity in CO oxidation applications. DFT calculates that the Cu atoms on the surface are anchored by the defective -OH or -OH_2_ ligands on the zirconia clusters. Zhao and coworkers also proved that defects in MOF help anchor metal atoms to prepare high-quality single-atom catalysts (Y. Zhao et al., 2019b).

The above studies explored that MOF is an ideal single atom catalyst support with many potential coordination sites and defects to anchor metal atoms. Similar to zeolites, MOF also has regular pores, which can confine metal atoms to prevent their aggregation. MOF confined single-atom catalysts are shown in electrocatalysis [such as oxygen reduction reaction (ORR) (Zhou et al., 2021), oxygen evolution reaction (OER) (Zhao et al., 2016), CO_2_ reduction reaction (CO_2_RR) (Cui et al., 2020), etc.] and photocatalysis (Fang et al., 2018; Guo et al., 2020) with excellent activity, selectivity and stability. However, current single-atom catalysts also have some problems with poor conductivity.

## Conclusion and Outlook

In this paper, we have reviewed the recent progress in the synthesis, characterization and catalytic application of SACs confined in porous materials, and discussed the possible catalytic mechanism. Porous materials, namely zeolites, carbon nitride (CN), and metal organic frameworks (MOF), were discussed. Among them, carbon materials are the most studied by researchers, usually only as a support, on which the appropriate type and quantity of main group elements (such as C, N and O) are necessary for the stability of single atom and the maintenance of their coordination unsaturated state. Zeolites and MOF are a kind of porous material with abundant pore structure and regular pore structure. Because of their advantages such as large specific surface area and high hydrothermal stability, they have become important support materials for confined single-atom catalysts. Other porous material confined single-atom catalysts also have great application potential.

Although many achievements have been made in the confinement of single-atom catalysts in porous materials. There is still a lot of work to be done by researchers. Innovative preparation methods and synthesizing high-performance thermally stable single-atom catalysts are conducive to broadening its application range.

In addition, high-quality single-atom catalysts confined in porous materials have great significance for practical applications. The catalytic mechanism of single-atom catalysts is different from traditional catalytic mechanisms, and a thorough study of single-atom catalytic mechanisms will help promote the development and progress of the catalytic industry. At present, the application fields of single-atom catalysts are mainly concentrated in electrocatalysis, photocatalytic hydrogen evolution, CO_2_ reduction and other reactions. It can be broadened to environmental pollution control (such as air pollution, water pollution treatment, etc.), petrochemical and other fields, and further develop the potential of single-atom catalysts.
